# Numerical Simulation of Monitoring Corrosion in Reinforced Concrete Based on Ultrasonic Guided Waves

**DOI:** 10.1155/2014/752494

**Published:** 2014-06-12

**Authors:** Zhupeng Zheng, Ying Lei, Xin Xue

**Affiliations:** Department of Civil Engineering, Xiamen University, Xiamen 361005, China

## Abstract

Numerical simulation based on finite element method is conducted to predict the location of pitting corrosion in reinforced concrete. Simulation results show that it is feasible to predict corrosion monitoring based on ultrasonic guided wave in reinforced concrete, and wavelet analysis can be used for the extremely weak signal of guided waves due to energy leaking into concrete. The characteristic of time-frequency localization of wavelet transform is adopted in the corrosion monitoring of reinforced concrete. Guided waves can be successfully used to identify corrosion defects in reinforced concrete with the analysis of suitable wavelet-based function and its scale.

## 1. Introduction

Civil infrastructures frequently cause failure due to corrosion of steel and rebar in concrete structures. Due to corrosion billions of US dollars should be spent annually in repair, rehabilitation, and reconstruction efforts of reinforced concrete structures. The fact makes it arguably the single largest infrastructural problem facing the industrialized countries [1]. Thus, it is very important to develop effective corrosion monitoring technologies. A wide range of techniques have been reported in the paper that can be employed for the monitoring of corrosion of steel in concrete structures for the purpose of diagnosing the cause and extent of the reinforcement corrosion [2]. Most of the current techniques are based on electrochemical methods such as half-cell potential mapping linear polarization. These techniques relate corrosion rate and extent through assessment on surrounding concrete medium. While many electrochemical techniques have been well established, none of these techniques concentrate on monitoring through direct condition assessment or measurements on embedded steel. As alternative tools for monitoring steel corrosion, some physical based techniques have been proposed [3, 4]. Compared with the electrochemistry based approaches, these physical approaches can not only provide supplemented tools for monitoring steel corrosion, but also conduct more accurate condition assessment of steel corrosion. Recently, the authors presented a review of some physical based monitoring techniques for condition assessment of corrosion in reinforced concrete in the past decades [5].

Among the current available physical monitoring techniques, the technique based on ultrasonic guided wave (UGW) is popular due to the advantages for monitoring corrosion related damage in reinforcing bars, so it has gained popularities in the recent years [5]. However, one difficulty of guided wave based technique for monitoring corrosion in reinforced concrete is the limitation of monitoring range for certain modes and frequencies [5, 6]. Unlike guided wave propagation in other multilayered systems, such as a metal pipeline in air, wave energy in steel bars embedded in mortar or concrete is lost (i.e., attenuated) at high rates due to leakage into the surrounding concrete. For the defects test of steel bar embedded in concrete, the reflected signals will be very weak, so the general time-frequency methods have difficulty in extracting the weak reflection signals of the defects in the detection signals. Meanwhile, there are many interference factors in the process of the experiment, for example, noise and the ideal boundary conditions which are difficult to achieve, and so forth, so it is hard to extract effective information of damage or defect from the received signals using guided wave methods. It is necessary to first investigate the problem by numerical simulation.

There are two methods widely used for the numerical simulation [7]. One is finite element method (FEM) and the other is boundary element method (BEM). BEM has been used in the wave guide of slab; for example, Cho and Rose [8] analyzed the mode conversion of Lamb wave at the reflection of boundary by BEM; Zhao and Rose [9] explored the guided waves on the identification of the size of defect by simulating various size and depth of defects on the slab using BEM. FEM has been mostly used in the wave guide of tube; for example, Demma [10] showed that a series of models of pipe with defects was calculated using FEM and the results were consistent with the experimental results. Moser et al. [11] simulated the propagation of elastic wave in the sheet and tubular structure using FEM. The results are fully consistent with those from experiment, which further proves the validity of the simulation in wave propagation using FEM. Cheng [12] used shell element to simulate the defect monitoring by longitudinal guided wave and get the relation curves between reflection coefficient and circumferential length or axial length of the defect in pipe. He et al. [13] studied the propagation of guided waves in bending pipe using FEM.

However, most of the previous analyses concentrated on thin wall pipe or slab using shell element in the simulation of guided waves. The shell element can only be used to simulate the pipes with very thin walls and is not suitable for the modes of guided waves with larger radial displacements [8]. There are few simulations for the rod structures. Therefore, it is proposed to explore the numerical simulation of corrosion monitoring of steel bar in reinforced concrete in this paper.

## 2. Numerical Simulation of Corrosion Monitoring in Reinforcement

When the ultrasonic guided waves are excited at the end of the steel bar and propagate along the axial direction of it, the mutation on the section dimensions or material properties of the steel bar will cause the strong discontinuity of the propagation of guided waves [14], resulting in reflection signals of guide waves from defects in the damage location. By the time process features of guided wave signals for the incidence, reflections from defects, and the end using sensors, the damage identification and location can be achieved.

The corrosion productions of steel are Fe(OH)_2_, Fe(OH)_3_, or Fe_3_O_4_, which are floc and have no strength. The pitting corrosion will cause the reduction in the section area of the steel bar, which is like notches formed in the surface of steel bar. Though there is difference in the geometry between the artificial defect (i.e., notch) and real corrosion in the steel bar, the research results [15, 16] show that the reflections for the mode L(0, 1) are almost the same for the artificial defect and real corrosion defect under the conditions of the same depth and circular length because the axial dimensions of these two defects are much shorter than the wavelength of mode L(0, 1). Therefore, the pitting corrosion is simulated by notch fabricated in the surface of the steel bar in this paper.

### 2.1. Modeling and Meshing

When the numerical simulation of guided waves is conducted using finite element method, the first step is to establish finite element model of the simulation component. According to the types of the established models, the finite element models for guided wave detection can be divided into the following three types: plane axial symmetric element model, shell element model, and entity unit model. These three models simplify respectively the actual waveguide in different way, which results in the differences of the rationality and the complexity for the established models [10]. This paper focuses on rod components, so the shell element model is not applicable and only the plane axial symmetric element model and entity unit model can be employed. In order to save the amount of computation, the plane axial symmetric element model is employed when the calculated results are better for the model with axial symmetry properties; otherwise, the entity unit model is used.

When establishing the finite element model, meshing is a very important step, and there is a high requirement on the mesh division for the finite element simulation of guided waves, in which the establishment of defect in the simulation model of the guided wave is one of the important issues. There are two main methods for modeling defect. The first one is establishing directly the component model of rod with defect and then meshing. In this way, defect can be simply established and various types of defects can be done, but the existence of defect destroys the regularity of the model and it is very difficult to mesh the model by mapping because the size of the defect is generally small. The other method is progressing as follows: firstly, establish a zero-defect model and map mesh and, then, remove the unit at the defect position. In this way, the regular grids of model can be achieved but the geometry of defect will be restricted by the element shape. In order to get regular girds, the latter method is adopted in this paper.

For the processing of boundary conditions, rigid boundaries are set at both ends of the cylinder steel bar [17]. At the same time, the axial boundary of concrete is set as no reflection boundary to make the result more reasonable in which uncertainty factors in simulation are eliminated, because the steel bars are distributed along the longitudinal direction of the concrete components in the actual on-line detection and there is no reflection on the border for the propagation of stress wave in concrete.

### 2.2. Signal Loading and Postprocessing

**Figure 1:**
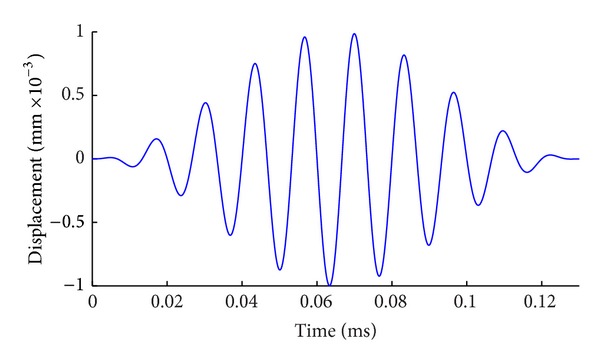
Excitation signal (center frequency: 75 KHz).

Research shows that guided waves can be excited in the waveguide when instantaneous displacement is loaded in all nodes of certain section in the model [18]. The incentive signal with the center frequency of 75 KHz is used in this paper for numerical simulation. The time-domain waveform of the signal is shown in Figure 1.

Numerical simulation of guided waves in this paper is the transient dynamic analysis. The excitation of guided waves is completed when axial displacement in the time history curve as shown in Figure 1 is applied in the left side of steel model as “signal-loading.” In postprocessing, time-displacement curve will be extracted and analyzed from the nodes at the receiving location and the data of corresponding results will be outputted to a text file for later analysis and processing.

### 2.3. Parameter Selection

It is the subject of transient elastic dynamic problems for the propagation of guided waves which is discrete by the finite element method in time and space domain. There is important significance for the selection of related parameters on the correctness of the results, especially the length of space discrete unit and time step of the discrete time. The main parameters involved in this paper are material, geometric, discrete, and incentive parameters, respectively. The material parameters are elastic modulus, Poisson's ratio, and density which are related to the waveguide shown in Table 1.

**Figure 2:**
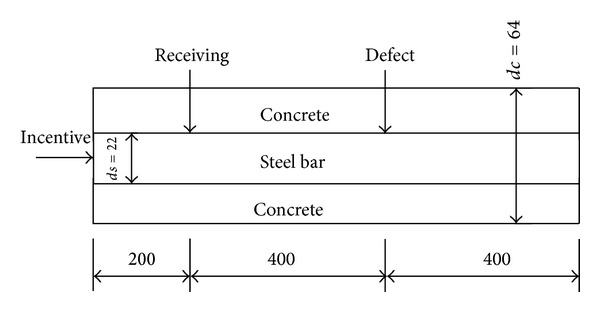
Layout of incentive and receiving (unit: mm).

Geometric parameters depend on the model, which are mainly including the diameter and the length of the component as well as locations of incentive and receiving, as shown in Figure 2.

Discrete parameter is one of the most important parameters in finite element simulation of guided wave and determines the precision of the finite element model. There are unit length and time step included in the parameters, which should be suitable for the small calculation error and enough precision. Moser et al. [11] proposed that there are at least 20 units needed in each wavelength of guided wave in the propagating direction and time step should be less than 0.8 times of the required time that guided wave propagates through a unit. The expressions are shown in Table 2, where *v* is the speed of the guided wave propagating in the waveguide. According to Table 2, the axial length unit takes 2 mm and time step takes 0.1 *μ*s in the model in this paper.

Incentive parameters mainly involve the mode and the frequency of excitation. Axial symmetric longitudinal modes are commonly used in the actual detection.

### 2.4. Excitation and Receiving of the Longitudinal Mode of Guided Wave in Reinforced Concrete

From the basic theory of guided wave, the axial order number for axial symmetric mode of guided wave is zero; consequently, the excitation and receiving are also axially symmetric. There are only the axial and radial displacements, no circumferential displacements for axial symmetric longitudinal mode. Research results show that the longitudinal mode of guided waves can be excited in the waveguide when instantaneous axial displacement is loaded in all nodes of certain section in the model [19]. In order to eliminate the influence of flexural modes, axial displacements of all the nodes at the receiving location are added up and the better axial symmetric longitudinal mode is obtained. According to this method, the axial symmetric longitudinal mode is excited and received in the reinforcement, and the layout is shown as Figure 2.

**Figure 3:**
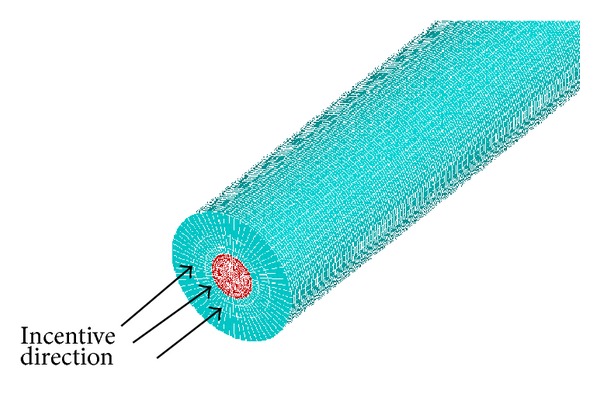
Scheme of axial symmetric longitudinal modal incentive.

According to the dispersion curve of group velocity for the steel bar, the sine signal modulated by Hanning window with ten cycles at the center frequency of 75 KHz is applied to all the nodes at the incentive location, which is along the axial direction of the bar. The model is shown as Figure 3.

**Figure 4:**
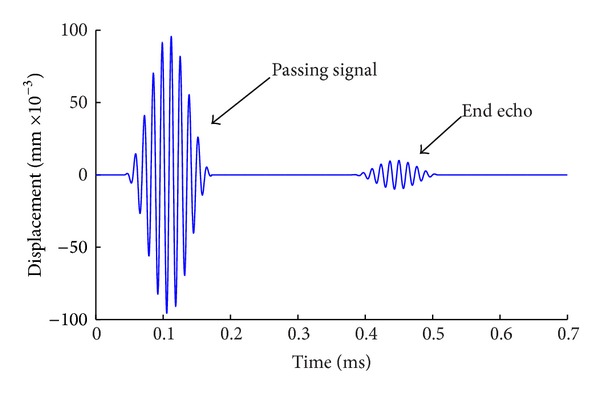
Waveform at receiving place after summation of all nodes axial displacement.

After solving, add up the axial displacement of all nodes at the receiving location. The results are shown in Figure 4. From the curve of displacement versus time in Figure 4, it can be seen that there are two groups of sine signals with ten cycles which are the passing signal and end echo signal, respectively. The propagation distance of guided wave from passing signal to echo signal is 1600 mm, and the time interval is 0.3326 ms. Therefore, the velocity of the wave is calculated as 4810 m/s. From the dispersion curve of group velocity of the steel bar, it is known that there are only the modes L(0, 1) and F(1, 1) at the frequency of 75 KHz and the velocity of mode L(0, 1) is 4745 m/s, which is almost the same as the velocity from the above calculation. The results show that the axial symmetric longitudinal mode L(0, 1) of guided waves can be excited in the steel bar by applying instantaneous displacement on the end of it and taking the axial displacement of nodes at the receiving location. It is also seen from Figure 4 that there is no other mode of waves besides the passing and echo signals, which show that the flexural mode F(1, 1) can be eliminated to get the pure mode L(0, 1) from the summation of displacement of nodes at the receiving location.

### 2.5. Pitting Corrosion Simulation

As mentioned earlier, the reflection signal of notch is the same as that of actual pitting corrosion, so the steel pitting corrosion is simulated by removing the unit at the corrosion location in the numerical simulation. Based on the established model, the member of reinforced concrete and the locations of defect and receiving position are designed as shown in Figure 2, which can avoid the overlay signals between the reflection from defect and the end.

**Figure 5:**
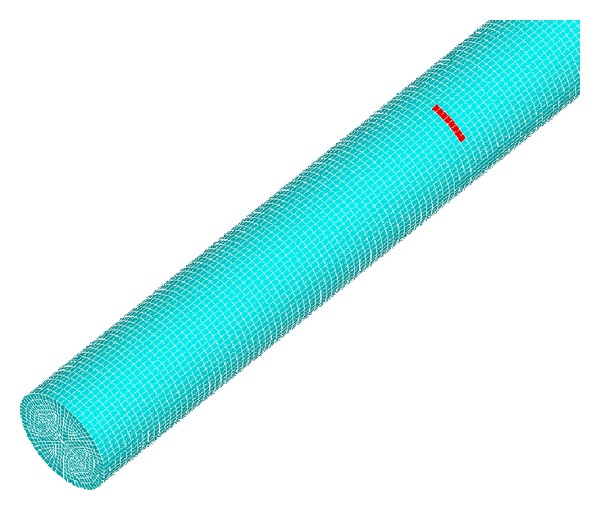
Finite element model of steel bar with defect.

The dimensions of defects are 2 mm in width and 1/8~1/2 times the perimeter in circumference length. The circumferential direction of the component is divided into 16 units and 4 units in radial direction. In this model the total number of units and nodes is 214292 and 315556, respectively. It takes 5 hours to work in the laboratory computer. The extraction model of steel bar from concrete is shown in Figure 5.

### 2.6. Simulation Results

**Figure 6:**
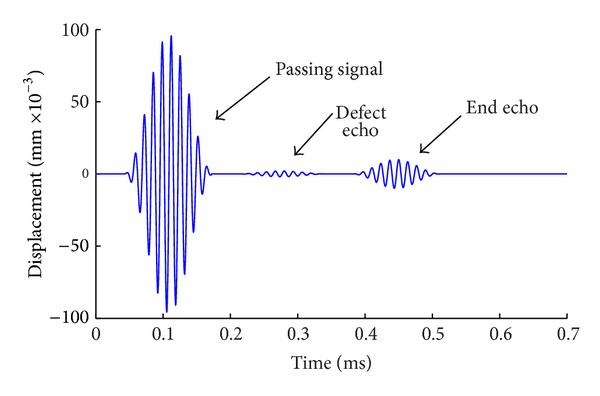
Detection signal with length of defect = 1/2 circumference.

**Figure 7:**
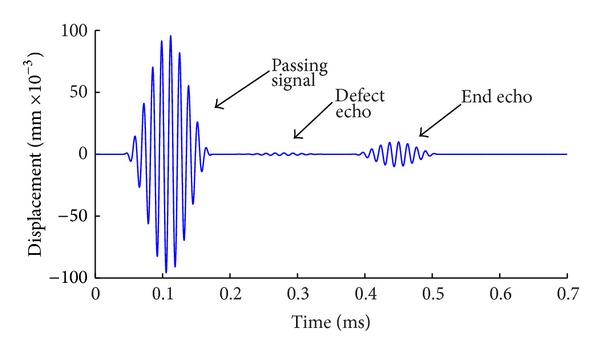
Detection signal with length of defect = 1/4 circumference.

**Figure 8:**
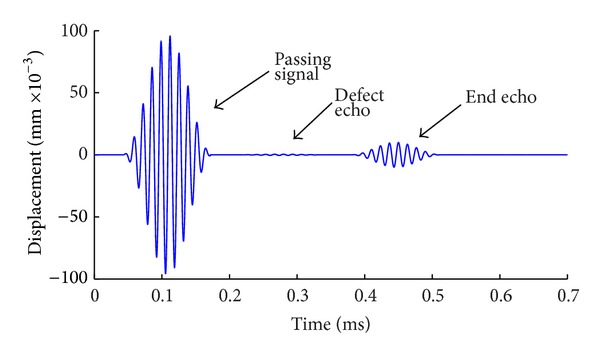
Detection signal with length of defect = 1/8 circumference.

The method is used to simulate the propagation of guided waves in the model with defect. Varying degrees of pitting are simulated by removing the steel units in the finite element model. Results are shown as Figures 6, 7, and 8. From Figure 6, the defect echo and end echo can be obviously identified. The damage location can be determined by the velocity of mode L(0, 1) at the frequency of 75 KHz and the propagating time of echo, which is the time interval between passing signal and the defect echo, that is, 0.206 ms in this case. Thus, the total propagating distance of defect signal is as follows:

(1) $$\begin{array}{c} S=C\times t=4745\times 2.06\times 10^{-4}=0.977\;\text{m}=977\;\text{mm}. \end{array}$$

The receiving location is 200 mm far from the left end of steel bar, so the location of defect *x* is known as

(2) $$\begin{array}{c} x=\frac{S+200}{2}=588\;\text{mm}. \end{array}$$

**Figure 9:**
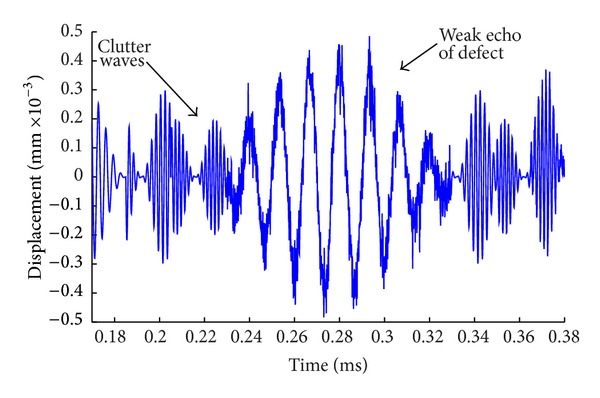
Amplification of the original signal.

Compared with the exact location of the defect in the model, the calculation results are consistent with those of the preset defect (error is within 2%). Results show that it is feasible to simulate the detection of guided waves in reinforced concrete by FEM and the built model is correct. However, it is difficult to identify the reflection signal of defect from Figure 8, because the signal is extremely weak and intertwined with other forms of faint waves. As shown in Figure 9, by amplifying directly the signals between 0.17 ms and 0.38 ms in the time domain, it can be seen that the refection wave is mixed together with a large number of narrow pulse type of clutter waves and the waveform is extremely too complex to distinguish the real reflection signal. Therefore, more detailed requirement in time domain or frequency domain is needed in adjusting the size of window of the function. Therefore the following method of wavelet analysis is to be used.

### 2.7. Identification of Weak Damage Signal by Wavelet Transform

When the guided-wave based technique is used to monitor corrosion defect in reinforced concrete, it is difficult to extract the weak reflection signal of corrosion defect from the detection signals by the general time-frequency method. At the same time, the background of collecting signals is influenced by various noises which is objective existence caused by the experiment environment. As a result it is difficult to intuit the effective identification of damage information from the detected signals. Wavelet transform is a kind of method for analyzing signal by 2 scales on time, which has the characteristics of multiresolution analysis and the very good characterization of local signal in time domain and frequency domain [20, 21]. Therefore special signals with certain characteristics (such as specific frequency or waveform) can be carried out by this method to be amplified and the extraction of signal detection and damage identification can be achieved.

In fact, wavelet coefficient obtained by wavelet transform is the correlation coefficient between the original signal with wavelet basis function in the corresponding scale and position which reflects the similarity between them [22]. Thus, it is a primary issue to select the appropriate wavelet basis function before wavelet transform because in essence it is the projection from the original signal to a set of wavelet bases which have influence on the results of wavelet transform. According to the characteristics of the wavelet basis function [23], during the selection of wavelet bases, the following factors are usually considered: the computing speed, attenuation of wavelet base, precise reconstructing of signal, presence of phase distortion, characteristics of filtering, time-frequency resolution, orthogonality and how to reduce the edge effect, and so forth. In the analysis of high order singular signal, the disappearance of the wavelet moment must be also considered. The wavelet is used in this paper for the analysis of signals of corrosion monitoring in the reinforced concrete, so the aspects of reasonable selection of wavelet basis function mainly include compact support, vanishing moment, regularity, orthogonality, and waveform.

There are a lot of wavelet functions available, such as Gaussian, Mexican Hat, Daubechies, and Biorthogonal. Because every wavelet function has its own structure and characteristics, the analysis results are also different and it is unadvisable to analyze all the different issues by the same wavelet function. The properties of wavelet function should be paid attention to in the application, which are directly related to the time-frequency localization, singularity detection, decomposition, and precision of reconstruction. Therefore, when wavelet analysis technique is engaged in damage identification for structural health monitoring, the wavelet function should be fit for detection of local mutation signal based on the features and purposes of analysis signals [24].

Based on the above principles and the similarity and power spectral matching between wavelet function and detection signals into account, this paper adopts Symlets wavelet to make the wavelet transform for the detection signals of corrosion in reinforced concrete, which is an improvement of the db wavelet proposed by Daubechies with orthogonality and compact support. Compared with db wavelet, there is significant improvement of symmetry in Symlets wavelet. The feature can avoid the distortion of signal in its decomposition and reconstruction.

**Figure 10:**
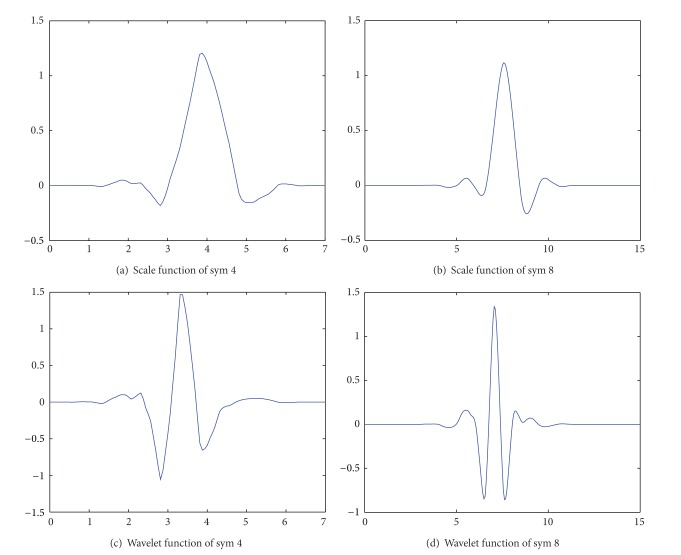
Wavelet function and scale function of sym 4 & sym 8.

Symlets wavelet is usually expressed as sym *N* (*N* = 2,3 … 8). When choosing wavelet function, the performance and the length of the filter should be considered so as not to affect the quality of waveform after wavelet transform [25]. Sym 1 wavelet cannot be used because its discontinuity of filter. Though the filter lengths of sym 2 and sym 3 wavelet are short, they are easily influenced by outside interference. The waveforms of scale function and wavelet function of sym 4 and sym 8 are shown in Figures 10(a)–10(d). It is found from the figures that not only the frequency characteristics of wavelet function and scale function of sym 8 are better than those of sym 4, but also the time domain resolution of sym 8 meet the requirements and its shape is more close to the actual curve. Therefore, the sym 8 wavelet with approximate symmetry, compact support, and biorthogonality is selected as the wavelet base to decompose the detected signal of corrosion monitoring in reinforced concrete.

One of the parameters to evaluate the characteristics of wavelet function is the scale, which is in an inverse relationship to the decomposition frequency of the transforming signal as shown in the following equation [26]:

(3) $$\begin{array}{c} F_{a}\times a\times \Delta =F_{c}, \end{array}$$

where *a* is the wavelet scale, Δ is the sampling period, *F*_*a*_ is the frequency of the transforming signal, and *F*_*c*_ is the center frequency of wavelet function and decided by the choice of wavelet function. Because the center of frequency window is related to the wavelet function, the relationship between the scale and the center frequency depends on the wavelet function and the sampling period.

It can be seen from (3) that the greater the scale, the lower the frequency resolution of the analysis signal, and the more characteristics gained in low frequency and vice-versa. The mother wavelet will turn into a set of wavelet basis functions after translation and scaling and then does the dot product with the signals to be analyzed under different scales. After that, the continuous wavelet transform will be achieved as shown in the following equation:

(4) $$\begin{array}{c} \text{CW}{\text{T}}_{x}\left(a,b\right)=\frac{1}{\sqrt{a}}\overset{+\infty }{\underset{-\infty }{\int }}x\left(t\right)\psi^{*}\left(\frac{t-b}{a}\right)dt, \end{array}$$

where CWT_*x*_(*a*, *b*) is the continuous wavelet transform, *ψ*(*t*) is the basic wavelet or named mother wavelet, ψ* is its conjugation, *x*(*t*) is the signal to be analyzed, *t* is the time, *a* is the scale factor, and *b* is the time shift parameter.

The wavelet transform is usually conducted using fast algorithms by the software of Matlab in the practical application. The remaining coefficient and wavelet coefficient in the space of scale *j* + 1 will be got after making weighted summation of filter coefficients on the basis of the remaining coefficient in the space of scale *j*, as shown in the following equations:

(5) $$\begin{array}{c} d_{j+1,k}=\underset{m}{\sum }h_{0}\left(m-2k\right)c_{j,m},\\ c_{j+1,k}=\underset{m}{\sum }h_{1}\left(m-2k\right)c_{j,m}, \end{array}$$

where *h*_0_(*n*), *h*_1_(*n*) are the filter coefficients, *d*_*j*+1,*k*_ and *c*_*j*+1,*k*_ are, respectively, the remaining coefficient and the wavelet coefficient in the space of scale *j* + 1.

After decomposition, the signals in the scale *j* are corresponding to the wavelet coefficients *c*_*j*,*k*_. The information of signals is complete and all components are preserved due to the integrity of wavelet space, which provides the analysis of the characteristics of the signal energy distribution.

In wavelet analysis, it is the first consideration of the range of wavelet scale and its division because it will lead to low recognition rate if the scale of wavelet is chosen blindly [27]. Therefore, in order to analyze the response signals of structure with target in detailed using wavelet transform, the range of analyzed signal frequency should be determined to get the corresponding scale range of wavelet function according to (3). Therefore the number and size of scales in wavelet transform will greatly affect the results of the identification.

**Figure 11:**
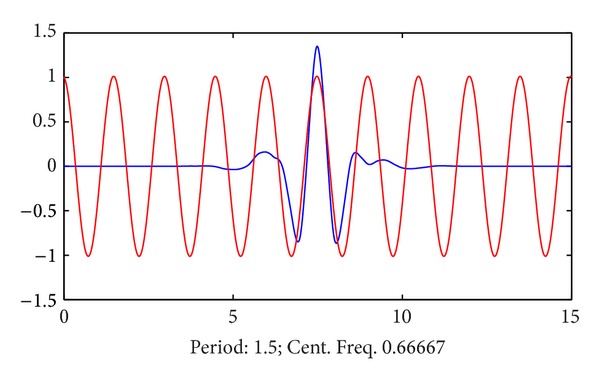
Waveform of sym 8 in time domain (shown in thick line).

First, the waveform of sym 8 wavelet in the time domain can be calculated using the file named centfrq.m in Matlab [27], which determines the center of frequency window. Results are shown in Figure 11, in which sym 8 wavelet is shown in thick line and its center frequency is around 0.667 Hz. The waveform of sine wave in the same frequency is also shown in fine line for comparison.

**Figure 12:**
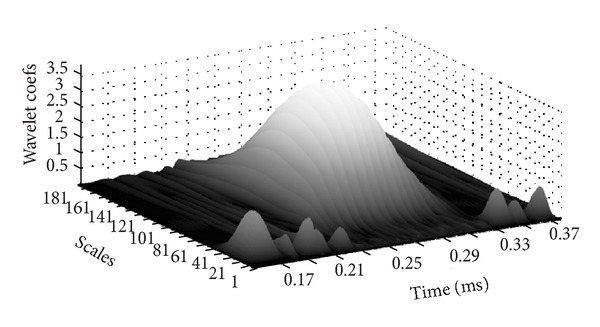
Wavelet transform of the detection signals at different scales.

In the case of unknown range of frequency in received signals, the number of scales of wavelet transform can be obtained by two ways. One is to estimate the range of signal frequency and then choose the step length of the analyzing frequency band. After dividing the range of frequency evenly, the size of scales of each band can be calculated from (3). The other way is to determine the size and number of the analyzing frequency band directly by evenly divided scales of wavelet, which will better reflect the band-pass filter function of wavelet transform. In this way, the energy characteristics of decomposed signal will be more obvious and the identification precision of signals can be significantly improved than the first one. Therefore, the evenly divided scales of wavelet are directly employed to get the number and size of analyzing band of signal frequency in this paper. The analysis range of the wavelet scales is 1 to 199, and the corresponding range of the signal frequency is 33.5 KHz to 6670 KHz which is consistent with estimated range. The step length of scale is 5; that is, the number of scale is 40. After wavelet transform of the signals in Figure 9, the results are shown in Figure 12.

After trial, it is found that it will make no contribution to improve significantly the identification precision of the signal by increasing the range of scales or decreasing the length of step, because it has met the need of signal analysis when the frequency range is corresponding to the scale range of wavelet. It will increase the number and the redundancy of detection signals and be unconducive to monitoring if the range of scales is increased or the length of step is decreased.

**Figure 13:**
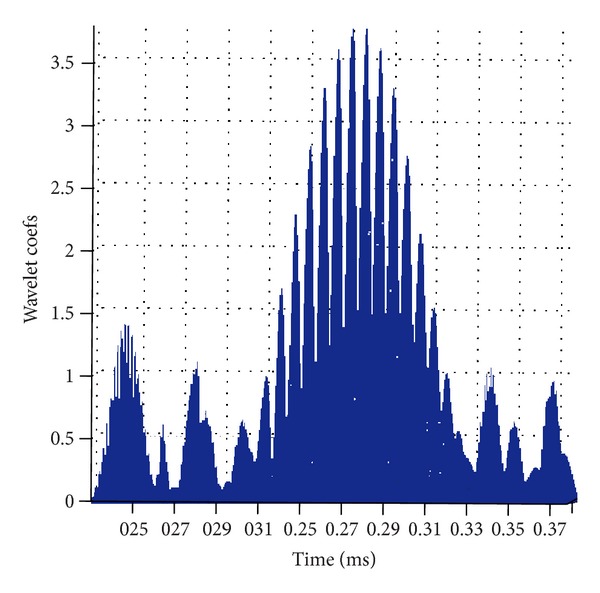
Detection signals after wavelet transform at scale of 89.

There are different time scales for different signals (such as noise, clutter, and defect reflection signal), so the results of wavelet transform will be different due to the chosen scale. In order to reflect the maximum similar degree between wavelet basis function and the defect reflection signal, that is, to get the maximum wavelet coefficient of defect reflection signal in the same scale, the corresponding scale can be obtained by the conversion of (3), as shown by

(6) $$\begin{array}{c} a=\frac{F_{c}}{\left(F_{a}\times \Delta \right)}, \end{array}$$

where, sampling period Δ refers to the time step in numerical calculation, which is 0.1 *μ*s in this case. *F*_*c*_ and *F*_*a*_ are the center frequency of wavelet function and telltale signal (i.e., defect echo), respectively, which are 0.667 Hz and 75 KHz, respectively, in this case. Thus the corresponding scale is 88.93 after calculation. Therefore, the scale of 89 is chosen and the waveform of wavelet transform to reflect the defect reflection signal is shown in Figure 13.

From the comparison of the results in Figure 13 with those in Figure 9, the defect reflection signal at the time of 0.28 ms from the detection signals which is obtained using appropriate scale by wavelet transform method can be clearly seen. The time of 0.28 ms is the same as the flight time that the echo of preset defect is needed, which proves that it is feasible and effective of the proposed method.

## 3. Conclusions

In this paper, the application of guided waves in monitoring corrosion of reinforced concrete is numerically simulated by finite element method. The methods for model establishment and critical parameters are analyzed by comparing the characteristic of each model. The approach for exciting and receiving guided wave with the longitudinal axial symmetric mode in finite element simulation is provided. Based on the preliminary numerical testing results, the pitting corrosion in the steel bar is simulated. Wavelet analysis, which has the time-frequency localization characteristic, is applied for the detection of weak damage signal resulting from energy leaking into concrete. The corrosion defects in reinforced concrete can be identified by selecting suitable wavelet-based function and scale. The numerical simulation is validated by the satisfactory agreement between the simulation and actual results.

## Figures and Tables

**Table 1:** Material parameters of steel bar and concrete.

Parameter	Steel bar	Concrete
Poisson's ratio	0.2865	0.27
Density (kg/m^3^)	7932	2200
Modulus (MPa)	215000	22000

**Table 2:** Discrete parameters.

Parameter	Value
Unit length Δ*x*	Δ*x* ≤ λ/20
Time step Δ*t*	Δ*t* ≤ 0.8 × Δ*x*/*v*
